# Game‐Based Foreign‐Language Speech Rehearsal Improves Pitch Processing Beyond Speech Domain

**DOI:** 10.1111/ejn.70603

**Published:** 2026-07-06

**Authors:** Sari Ylinen, Katja Junttila, Anna‐Riikka Smolander, Reima Karhila, Mikko Kurimo

**Affiliations:** ^1^ Speech‐Language Pathology, Welfare Sciences, Faculty of Social Sciences Tampere University Tampere Finland; ^2^ Cognitive Brain Research Unit, Department of Psychology, Faculty of Medicine University of Helsinki Helsinki Finland; ^3^ Department of Information and Communications Engineering Aalto University Espoo Finland

**Keywords:** game‐based learning, mismatch negativity (MMN), phonetic training, pitch processing, second‐language (L2) learning

## Abstract

When training new skills, it is beneficial if they generalize to new items beyond those explicitly trained. In this study, we investigated whether game‐based training of second‐language speech sounds and words affects the processing of untrained sounds, specifically focusing on the pitch of nonspeech sounds. Thirty‐seven school‐aged children participated in a longitudinal study with two or three measurement points, during which mismatch negativity (MMN) responses were recorded using electroencephalography. The treatment group received 4–5 weeks of game‐based training between the first and second measurement sessions. The delayed‐treatment group waited for 4–5 weeks between the first and second sessions and then received the game‐based training, the effects of which were assessed in a third session. Results showed that MMN peak amplitude increased across measurement sessions. However, MMN latency decreased in the treatment group following the gaming period, whereas it increased in the delayed‐treatment group. After receiving the game‐based training, MMN latency decreased also in the delayed‐treatment group. This pattern of results suggests that the effects of second‐language speech training may generalize to the processing of untrained, nonspeech sounds. The observed improvement in pitch processing may be due to the link between pitch perception and phonological skills targeted by the game‐based training. Additionally, the gaming approach itself may contribute to the reduction in MMN latency. Taken together, the findings indicate that second‐language speech training can enhance neural processing of untrained auditory stimuli, thereby demonstrating cross‐domain auditory plasticity.

## Graphical abstract

In this longitudinal EEG study, game‐based second‐language (L2) speech‐sound training promoted generalization to untrained pitch changes in nonspeech sounds, as evidenced by decreased mismatch negativity (MMN) latency at the Fz site in children who received 4–5 weeks of training. In contrast, latency increased in the delayed‐treatment group until they later received the same training. The responses are deviant‐minus‐standard difference waveforms, and negativity is plotted upward.

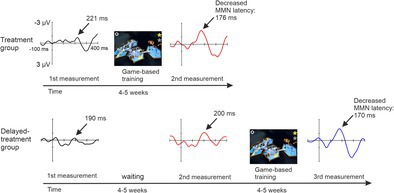

**Abbreviations**
ANOVA: analysis of variance
ASR: automatic speech recognition
CI: 95% confidence interval
dB: decibel
EEG: electroencephalography
ERP: event‐related potential
F0: fundamental frequency
L2: second language
MMN: mismatch negativity
ROI: region of interest
SD: standard deviation

## 1 Introduction

Languages have different phonological systems, and they may use different cues to signify phonological contrasts. Therefore, the early phases of second‐language (L2) learning include learning to perceive and produce the sounds of that language, which is needed to recognize and produce its words and grammatical structures. L2 speech‐sound learning requires both perceptual and articulatory rehearsal. Experimental studies on L2 perceptual or articulatory rehearsal typically focus on a specific phonetic contrast and provide targeted training for it to ensure its success (e.g., Bradlow et al. 1999; Logan et al. 1991; Lively et al. 1993, 1994; Ylinen et al. 2010; see Uchihara et al. 2025, for a recent meta‐analysis of high‐variability phonetic training).

Such studies have also examined how speech‐sound learning generalizes to new lexical items, asking whether learners can identify or discriminate the trained sounds in novel phonetic contexts, such as in words not included in the training (Bradlow et al. 1999; Carlet and Cebrian 2022; Lively et al. 1993, 1994; Uchihara et al. 2025). They have likewise investigated whether phonetic learning transfers to new talkers who were not included in the training phase (Bradlow et al. 1999; Logan et al. 1991; Lively et al. 1993; Uchihara et al. 2025).

Despite extensive research on generalization to new stimuli involving the trained speech sounds (Uchihara et al. 2025), only a very limited number of studies have addressed whether phonetic training generalizes to untrained features, including untrained speech sounds (for a review on auditory training and generalization effects in the nonspeech domain, see Wright and Zhang 2009). Tremblay et al. (1997) demonstrated that phonetic training of a prevoiced labial stop (/ba/) contrast can generalize to the processing of an untrained prevoiced alveolar (/da/) contrast. The results suggest that the effects of phonetic training can extend beyond the specific acoustic patterns used in training, but in both contrasts, the target feature of discrimination was the same, namely, prevoicing signaled by voice onset time (VOT). In another relevant study, Kurkela et al. (2019) exposed their participants passively to Chinese lexical tones for 8 h. Their results showed that passive exposure modulated event‐related potentials (ERPs)—mismatch negativity (MMN), P3a, and P3b—for spoken lexical tones. A modulation of MMN latency was also observed when nonspeech stimuli mimicking the tones were presented. In this setup as well, the target feature was identical during exposure and testing, namely, the contour of the fundamental frequency (F0).

To explore generalization using testing and training stimuli that differed more substantially in their features, our previous study (Ylinen et al. 2021) examined whether the effects of game‐based articulatory rehearsal of multiple novel L2 sounds in children learning English would generalize to untrained contrasts in two untrained languages, Mandarin and Russian. Specifically, the aim was to investigate whether the effects of extensive phonetic training of multiple L2 sounds generalize to new untrained items by increasing general sensitivity to speech sounds. The hypothesis of increased sensitivity was adapted from Simmonds' (2015) original proposal, which suggests that articulatory experimentation with new L2 sounds may enhance brain plasticity by shifting the brain into a learning state that resembles the song‐learning stages observed in songbirds. The results of Ylinen et al.'s (2021) study were somewhat ambiguous, showing clear improvement in the processing of a third‐language consonant contrast in some, but not all, children. This was suggested to reflect a lack of interest or motivation in the nonimprovers—not necessarily toward the speech training with the game but toward the test procedure including a behavioral auditory discrimination task, where responses were given by tapping the touch screen of a mobile device (for details, see Ylinen et al. 2021). In children, behavioral discrimination is prone to fluctuations in attention, motivation, and co‐operation. Therefore, generalization effects, if any, might be better found using electrophysiological measures revealing automatic processes of the brain, such as the MMN brain response (Näätänen et al. 1978).

The MMN is a component of auditory ERP that can be used as an index of auditory processing accuracy (for a review, see Näätänen et al. 2007; Näätänen and Kreegipuu 2012). Since the MMN is elicited by infrequent and thus unpredicted sounds in an auditory sequence with some regularity, it is considered a prediction error response, thus also reflecting predictive modelling of the sound environment in the brain (Winkler 2007). The MMN is elicited by changes in various acoustic features, including sound frequency, intensity, duration, and location (e.g., Näätänen et al. 2004; Näätänen et al. 2007). Typically, larger MMN amplitude and shorter latency reflect more accurate processing (e.g., Näätänen et al. 2007). Since MMN measurement does not require overt responses or even attention (Näätänen et al. 1978; Näätänen et al. 2007) to occur, it is a particularly useful method of study for children with fluctuating attention spans and levels of cooperation. It is also feasible in the study of linguistic and phonetic features (Näätänen et al. 1997; Pulvermüller and Shtyrov 2006). Importantly for the present study, MMN has been widely used to index brain plasticity and auditory training effects for both speech (Junttila et al. 2022; Junttila et al. 2023; Kraus et al. 1995; Tamminen et al. 2015; Tremblay et al. 1997; Ylinen et al. 2010) and nonspeech sounds (Gottselig et al. 2004; Kujala et al. 2003; Liu and Holt 2011; Perez et al. 2017), in both children (e.g., Junttila et al. 2022; Junttila et al. 2023; Kujala et al. 2001) and adults (e.g., Kraus et al. 1995; Tremblay et al. 1997; Ylinen et al. 2010).

Although training effects are typically stronger for trained than for untrained items (Wright and Zhang 2009), in some cases, training in one domain may yield broader, cross‐domain benefits; certain cognitive skills have been found to be interrelated, and training one may therefore influence the other. For example, previous studies have associated the processing of pitch in music or speech with phonological skills or phonological awareness both in children (Bolduc and Montésinos‐Gelet 2005; Loui et al. 2011; Steinbrink et al. 2019) and in adults (Skoe et al. 2017; Sun et al. 2017). Thus, extending the phonological system with speech training could be hypothesized to yield benefits for pitch processing beyond the speech domain, but the effects of such training on nonspeech processing are not known. Therefore, the current study aimed to explore whether L2 articulatory training improves the processing of pitch in nonspeech sounds beyond the trained speech domain. According to the hypothesis, the link between pitch processing and phonological skills (Bolduc and Montésinos‐Gelet 2005; Loui et al. 2011; Steinbrink et al. 2019) suggests that training designed to extend the phonological system may improve pitch processing across domains. Furthermore, it has been hypothesized that articulatory experimentation taking place in learning to produce new L2 sounds may increase general auditory plasticity in the human brain, similarly as during song learning in songbirds (Simmonds 2015). Potential training effects may be especially pronounced in children, given their heightened cortical plasticity for language (for a review, see White et al. 2013). The current study included children who were beginning learners of English or were about to start formal English instruction, to ensure that they could benefit from an L2 intervention targeting spoken English.

## 2 Methods

### 2.1 Ethics Statement

The study was approved by the University of Helsinki Ethical Review Board in the Humanities and Social and Behavioural Sciences, and it was conducted according to the Declaration of Helsinki.

### 2.2 Participants

The data were collected from the same group of 37 participants as reported by Junttila and colleagues' (2022) study, which compared game‐based and nongame language learning of L2 speech sounds and words (i.e., the research questions, datasets, and theoretical background of that and the current study were distinct). The participants were school‐aged Finnish‐speaking children (19 girls, 18 boys; mean age, 9 years 6 months; SD, 9 months; age range, 7 years 11 months–11 years 2 months). They were recruited through schools in the Helsinki metropolitan area and studied in Grades 2–5. This age group was selected because the intervention involved a language‐learning game designed for children at the early stages of learning English. At the time the study was launched, English instruction in Finnish schools typically began in Grade 3, when children were approximately 9 years old. Thus, the participating children were either already studying English or about to begin formal English instruction and could therefore benefit from the L2 intervention. According to parental reports, 27 children were studying English at school, three regularly attended an English language club, and five had learned some English informally through travel or other sources. Two children were not explicitly reported to have prior knowledge of English; however, a parent of one of these children reported the child's exposure to English through videos and games. In addition, 25 children were reported to have some proficiency in another foreign language besides English (German, Swedish, Spanish, or French). All children attended public schools following the national core curriculum, which includes basic music instruction. Furthermore, 18 children either pursued music as a hobby or had previously attended a musical play school.

The inclusion criteria were as follows: according to parental reports, age between 7 and 11 years, being a monolingual native Finnish speaker, no developmental disorders, no language disorders, no learning disorders, no head injuries, normal hearing, and normal vision or vision corrected to normal with eyeglasses. In addition to parental reports, the children's cognitive, phonological, and literacy skills were assessed (see Junttila et al. 2022, 2023). As part of inclusion criteria, the children's cognitive skills were screened with Wechsler Intelligence Scale for Children IV (WISC‐IV; Wechsler 2010) subtests Block Design, Digit Span, Coding, and Vocabulary, where they had to fulfill the criterion of score of not below 1.33 standard deviations in each task (i.e., they needed to score a minimum of 6 standard points) to be included in the study. All participants gave informed oral consent, and their legal guardians gave informed written consent.

**FIGURE 1:**
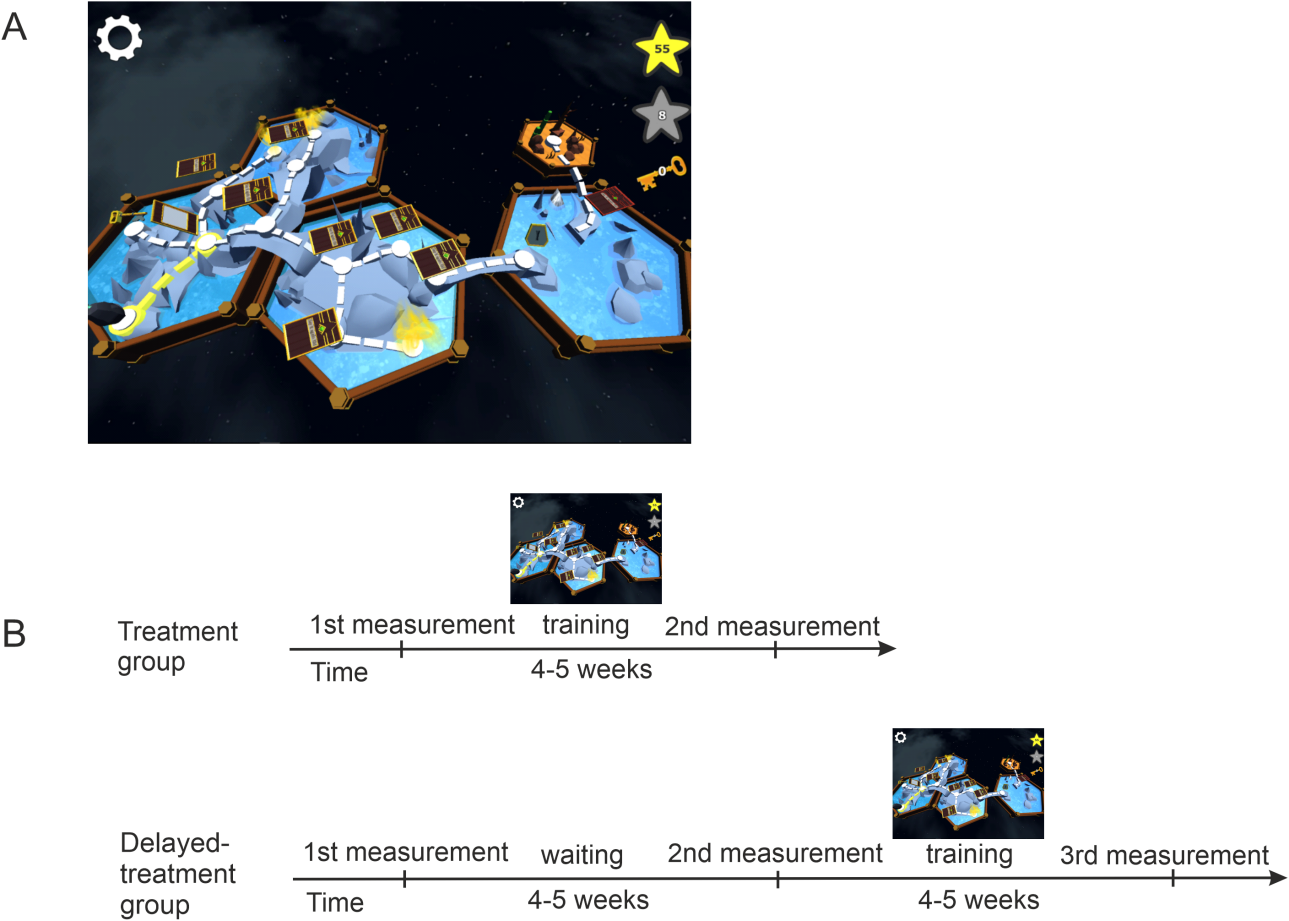
(A) A screenshot from the “Say it again, kid!” game used in the gaming intervention. (B) Setup of the study.

All participants took part in electroencephalography (EEG) measurements and a game‐based L2 intervention; however, the timing of the intervention varied, with a subset of children receiving it immediately and the remainder after a delay. First, a treatment group of 15 children (7 girls, 8 boys; mean age, 9 years 5 months) participated in the 1st and 2nd EEG measurement sessions and received the gaming intervention between them. Second, a delayed‐treatment group of 22 children (12 girls, 10 boys; mean age, 9 years 8 months) was tested in the same way as the treatment group, but they were placed on a waiting list. They received the gaming treatment after the 2nd measurement session and were tested again in the 3rd session (see Figure 1). During the intervention, children in the treatment and delayed‐treatment groups played the game for an average of 187 and 194 min, respectively. In the treatment group, 10 children studied English at school, and five had learned English during their free time (e.g., through an English language club). In the delayed‐treatment group, 17 children studied English at school, and three had learned English during their free time. Although two children in the delayed‐treatment group were not explicitly reported to have prior knowledge of English, a parent of one of these children reported exposure to English through videos and games.

### 2.3 Study Design and Stimuli

The auditory MMN brain responses to nonspeech sounds were measured with EEG before and after a gaming intervention in both groups (the 1st and 2nd measurement sessions for the treatment group; the 2nd and 3rd measurement sessions for the delayed‐treatment group) and before and after a waiting period (the 1st and 2nd measurement) without intervention in the delayed‐treatment group (see Figure 1). During EEG measurement, stimulus sounds were presented binaurally through headphones with a comfortable hearing level (60 dB). The sounds were presented in a passive oddball paradigm: The participants were presented with a sequence of repetitive standard sounds (80%) and occasional deviant sounds that differed from the standards with respect to their frequency (10%) or duration (10%). The current study focuses on the processing of the frequency change (for responses to duration, see Supporting Information). The stimulus presentation included one block of 800 auditory stimuli (640 standards, 80 frequency deviants, 80 duration deviants). Because the same recording session also included the study setup reported by Junttila et al. (2022), the total EEG recording time was approximately 2 h, including preparation and breaks.

The sounds used in the EEG experiment were harmonic tones that were clearly perceived as nonspeech. The standard stimuli in the oddball sequences had an F0 of 500 Hz, harmonic partials at 1000 and 1500 Hz, and a duration of 50 ms. The frequency deviant had an F0 of 550 Hz, harmonic partials at 1100 and 1650 Hz, and a duration of 50 ms, whereas the duration deviant had an F0 of 500 Hz, harmonic partials at 1000 and 1500 Hz, and a duration of 25 ms. The order of stimuli was pseudorandom: There were at least two standards between the deviants. Interstimulus interval (ISI, offset to onset) randomly varied between 250 and 350 ms.

### 2.4 Data Acquisition and Analysis

EEG was measured with BioSemi ActiveTwo system (BioSemi Inc., Amsterdam, The Netherlands) and BioSemi ActiView Version 7.07 EEG acquisition software using electrode caps with 64 channels and additional electrodes on the two mastoids to enable re‐referencing, outer canthi of the eyes to monitor horizontal eye movements, and under the left eye to monitor vertical eye movements. Nose served as the online reference. The sampling rate of the recording was 512 Hz. During the EEG recording, participants watched a self‐selected muted movie with Finnish subtitles. They were instructed to concentrate on the film and to ignore the auditory stimuli.

Data were further analyzed offline with BESA Research 7.0 software (BESA GmbH, Gräfelfing, Germany) and in‐house scripts for Matlab (MathWorks Inc., Natick, MA, USA). In BESA Research 7.0, default ocular artifact correction for vertical and horizontal eye movements was applied. Bad channels were interpolated (or rejected if interpolation was not possible; no more than five channels were interpolated per block). Then, data were filtered using a zero‐phase filter with a band pass of 1–20 Hz (slope 24 dB/octave), epochs of −100 to 400 ms were extracted from continuous EEG, and deviants and standards (excluding two standards following each deviant) were averaged separately for each measurement point. Artifacts exceeding ±100 μV were rejected from averaging. Baseline of the waveforms was corrected to zero at a 100‐ms prestimulus window. The data were re‐referenced to the average of the mastoids to improve the signal‐to‐noise ratio. On average, the number of accepted deviant trials was 76 (SD 3). The number of accepted deviant trials was comparable across groups (treatment: 77, delayed‐treatment: 75) as well as across sessions (1st session: 76.4; 2nd session: 75.4, 3rd session: 75.4).

To quantify the response latencies and amplitudes, deviant‐minus‐standard difference waves were formed. Time windows for MMN amplitude and latency measurement were derived from the grand‐average waveforms by identifying the most negative peak between 100 and 250 ms for each condition, since the MMN typically peaks in this time window (Näätänen 2001; see also Näätänen et al. 2007, for a notion of 150‐ to 250‐ms window, which is also compatible with the current data). Participants' amplitudes and latencies were then measured from individual difference waveforms by determining the amplitude and latency of the most negative peak in a 100‐ms time window centered around the grand‐average peak latency, determined separately for each condition (the time windows were 170–270 ms and 122–222 for the 1st and 2nd measurement sessions, respectively, in the treatment group and 143–243 ms, 151–251 ms and 127–227 ms for the 1st, 2nd, and 3rd measurement sessions, respectively, in the delayed‐treatment group). Amplitude and latency values were analyzed by averaging across the fronto‐central region of interest (ROI) including electrodes F3, Fz, F4, FC3, FCz, FC4, C3, Cz, and C4.

### 2.5 Gaming

Between the EEG sessions, the treatment‐group children participated in a game‐based training of common English words with the “Say it again, kid” (SIAK) language learning game (Karhila et al. 2017, 2019, 2023; Ylinen and Kurimo 2017; see Junttila et al. 2022, for an earlier description of the game intervention). In the game, players explored a game board with flashcards (see Figure 1). When opening a card, they saw a picture related to the target word, heard the word in Finnish, and then heard native English speakers' utterance of the word. The player was then prompted to say the word aloud. After this, the players heard once more their own utterances played back to them and the original English speaker's utterance for comparison.

Players were encouraged to produce the words accurately by awarding them with 1–5 stars according to their speech production accuracy. Players' utterances were assessed by automatic speech recognition (ASR) technology optimized for child speech (Karhila et al. 2017, 2019, 2023; for ASR embedded in a game, see also Getman et al. 2023). The collection of stars was required to proceed in the game. At the end of each level, the players' learning was tested with a test card where the learned words were produced without a model. Feedback provided by ASR was expected to support and reinforce learning and to motivate children to try their best to improve their pronunciation.

The gaming period lasted 4–5 weeks. On average, the treatment group played the game 12.5 min per day for 15 days spread over 29 days (mean total time, 187 min). After the waiting period between the 1st and the 2nd EEG session, the delayed‐treatment group played the game 17.5 min per day for 11 days spread over 31 days (mean total time, 194 min).

### 2.6 Statistical Analysis

The statistical significance of amplitude and latency effects in the fronto‐central ROI (averaged across electrodes F3, Fz, F4, FC3, FCz, FC4, C3, Cz, and C4) in the two groups was tested with the analysis of variance (ANOVA) with group (treatment vs. delayed treatment) as a between‐subject factor and test session (1st vs. 2nd) as a within‐subject factor.

The statistical significance of the effects in the delayed‐treatment group across their three test sessions was tested with the ANOVA with test session (the 1st vs. the 2nd before intervention vs. the 3rd after intervention) as a within‐subject factor. Effect sizes were reported as partial *η* ^2^. The effect sizes for pairwise comparisons following interactions were reported using Cohen's *d*. In addition, to further examine the relationship between the treatment and the MMN findings, Pearson's *r* correlations were calculated between the time spent on game‐based training and changes in the MMN (i.e., amplitude and latency differences between the pretreatment and posttreatment sessions).

To assess the sensitivity of our analyses with respect to sample size, we conducted a post hoc sensitivity analysis using G*Power (version 3.1.9.7; Faul et al. 2009). For the between‐subjects analysis of participants who received the treatment (*N* = 37; 2 groups × 2 time points), using *α* = 0.05 and a desired power of 0.80, the design was sensitive to detecting effects of *f* = 0.24 or larger. For the within‐subjects analysis of the delayed‐treatment group (*N* = 22; one group with three repeated measurements), using *α* = 0.05, power = 0.80, an assumed correlation among repeated measures of 0.50, and *ε* = 0.75, the minimum detectable effect size was *f* = 0.25. These detectable effect sizes fall within the medium range, indicating that the study had adequate sensitivity to detect effects of practical and theoretical relevance.

## 3 Results

**FIGURE 2:**
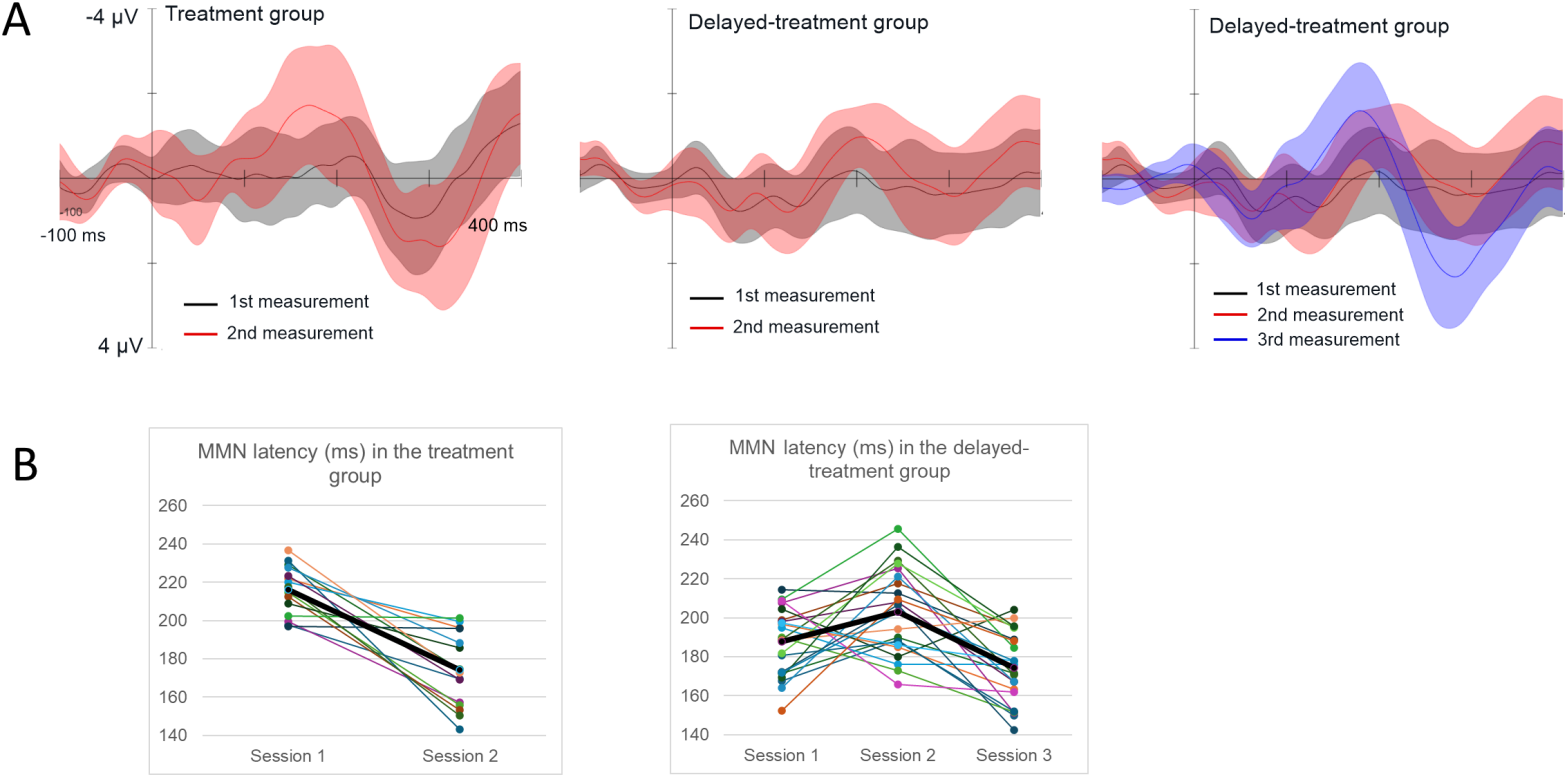
(A) Deviant‐minus‐standard difference waveforms showing the MMN responses to pitch change across measurement sessions, averaged over the fronto‐central region of interest (ROI). Shaded areas denote 95% confidence intervals for each waveform. Negativity is plotted upward. (B) MMN latencies of individual participants (thin lines) and the group average (thick lines) across two or three measurement sessions for the treatment and delayed‐treatment groups, respectively.

**FIGURE 3:**
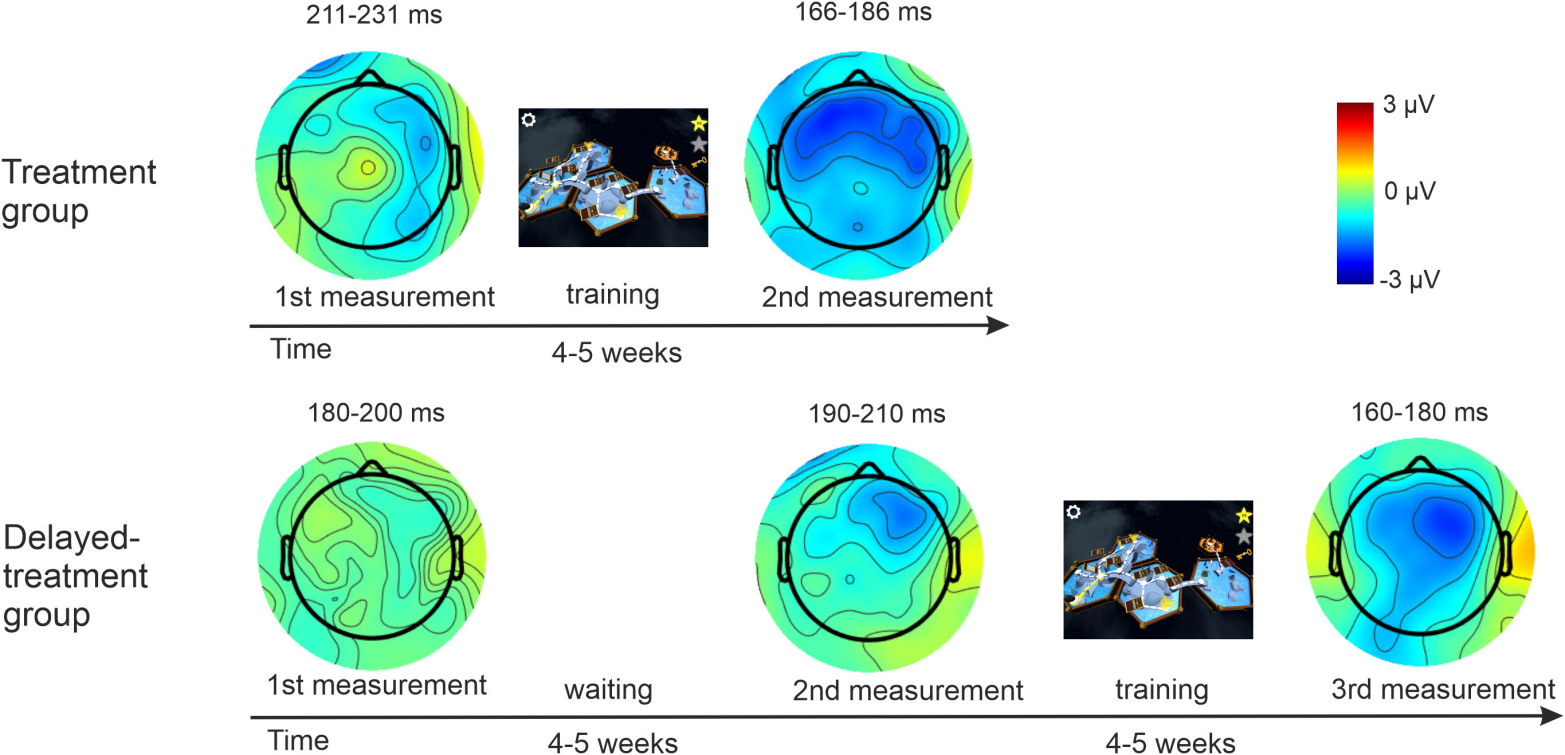
Scalp maps as a function of time across the groups and measurement sessions. The latency window of the scalp maps (20 ms centered at the Fz peak latency) has been given above each map. To conceptualize the electrode locations, Fz is located one 20% step above Cz along the midline, whereas Cz is located at the vertex (i.e., at the midpoint between the nasion and the inion, and the left and right preauricular points).

The MMN responses at fronto‐central ROI are illustrated in Figure 2 and scalp topography in Figure 3 (for original ERP responses to standards and deviants and the MMN responses to duration deviants, see Supporting Information). Analysis of MMN peak amplitude showed that amplitude was significantly larger in the 2nd measurement (−2.70 μV) than in the 1st measurement (−1.06 μV), as indicated by a main effect of test session [*F*(1,35) = 13.97, *p* < 0.001, partial *η* ^2^ = 0.29]. No significant interaction with group was found [*F*(1,35) = 0.43, *p* = 0.52, partial *η* ^2^ = 0.01]. MMN latency differed significantly between the first and second sessions, as indicated by a significant main effect of test session [*F*(1,35) = 8.24, *p* = 0.007, partial *η* ^2^ = 0.19]. However, this effect was in more detail explained by a significant interaction between group and test session [*F*(1,35) = 38.42, *p* < 0.001, partial *η* ^2^ = 0.52]. According to follow‐up pairwise comparisons, in the treatment group, the MMN latency was significantly shorter in the 2nd session (174 ms) than in the 1st session (216 ms; *p* < 0.001, Cohen's *d* = −1.67), whereas in the delayed‐treatment group, an opposite effect was observed, that is, the latency was significantly longer in the 2nd session (203 ms) than in the 1st session (188 ms, *p* = 0.013, Cohen's *d* = 0.53; see Figure 2).

Although the delayed‐treatment group children were originally assigned to a waiting list, they participated in the gaming intervention after the 2nd test session, and their brain responses were then again measured in the 3rd session. Statistical analysis of the MMN peak amplitude indicated a significant main effect of Test session [*F*(2,42) = 4.15, *p* < 0.023, partial *η* ^2^ = 0.17] due to increasing amplitude as a function of sessions (1st to 3rd session: −0.88, −2.29, and −2.46 μV, respectively). Further Bonferroni‐corrected pairwise comparisons were, however, not significant (for 1st vs. 2nd session, Cohen's *d*: −0.50; for 1st vs. 3rd session, −0.53; for 2nd vs. 3rd session, −0.06). Similarly, the analysis of MMN latency showed a significant main effect of test session [*F*(2,42) = 13.73, *p* < 0.001, partial *η* ^2^ = 0.40]. Unlike with the amplitude, however, Bonferroni‐corrected pairwise comparisons indicated significantly shorter latency (174 ms) in the 3rd session as compared with the 1st (188 ms, *p* = 0.042, Cohen's *d* = −0.57) and 2nd (203 ms, *p* < 0.001, Cohen's *d* = −1.19) test sessions (see Figure 2). In the 1st and the 2nd test sessions (i.e., before and after the waiting period, 188 vs. 203 ms, respectively), the latencies did not differ from each other significantly (Cohen's *d* = 0.53). The mean fronto‐central peak amplitudes and latencies across sessions and groups are provided in Table 1.

**TABLE 1:** The MMN amplitude (μV) and latency (ms) across the measurement sessions in the treatment and delayed‐treatment groups, averaged across fronto‐central scalp sites. Each value is followed by standard deviation (SD) and 95% confidence interval (CI).

	Amplitude in μV (± SD), CI			Latency in ms (± SD), CI		
	1st session	2nd session	3rd session	1st session	2nd session	3rd session
Treatment group	−1.31 (1.57) [−2.18 to −0.44]	−3.32 (2.43) [−4.66 to −1.97]	—	216 (13) [209–223]	174 (19) [163–185]	—
Delayed‐treatment group	−0.88 (2.24) [−1.88 to 0.11]	−2.29 (1.72) [−3.05 to −1.53]	−2.45 (2.59) [−3.60 to −1.31]	188 (17) [180–195]	203 (22) [193–213]	174 (18) [166–182]

The correlation analysis for training time and MMN changes between the pretreatment and posttreatment sessions showed no significant correlations (amplitude: *r*(37) = −0.01, *p* = 0.95; latency: *r*(37) = −0.08, *p* = 0.64).

## 4 Discussion

The current study investigated whether playing a speech‐based language learning game affects pitch processing beyond the speech domain. The MMN amplitude to a frequency change in nonspeech sounds increased between test sessions but did not interact with group. However, the MMN latency for these sounds decreased significantly from the 1st to the 2nd test session in the treatment group but increased in the delayed‐treatment group. A similar significant decrease of the MMN latency was observed in the delayed‐treatment group after they got the treatment.

The pattern of results suggests that in the current setup, the MMN amplitude increased between the test sessions. There was no significant interaction between the groups: the amplitude changed not only in those who completed game training between their two measurement sessions but also in participants with delayed treatment who received no training between the 1st and the 2nd sessions. This suggests that game training was not the sole factor underlying the increase in amplitude. Rather, the results suggest that the MMN amplitude was at least partly modulated by the repeated test sessions, that is, the study design of repeated measurements has likely affected the MMN amplitude. When comparing the MMN amplitude across the three recording sessions in the delayed‐treatment group, the largest increase appears to take place between the 1st and 2nd sessions, and further exposure in the 3rd session has only a small effect. Although a significant increase in amplitude across test sessions was observed when the two groups (*N* = 37) were analyzed together, and a significant main effect of test session was also found in the ANOVA for MMN amplitude in the delayed‐treatment group, the pairwise comparisons following this main effect did not reach significance. This suggests that the pattern of amplitude increase was not fully consistent within the group of 22 delayed‐treatment participants.

No similar increase of MMN amplitude was observed in our earlier studies using the same kind of repeated‐measurement study design before and after gaming but speech stimuli instead of nonspeech sounds (Junttila et al. 2022, 2023). Possible explanations for this discrepancy are the complexity of stimuli and the acoustic saliency of their differences: simple harmonic tones with a salient 500‐Hz difference in the current study in contrast with natural spoken L2 words with subtle acoustic differences in the earlier studies. Simple and salient tones may be more prone to auditory perceptual learning from exposure within a recording session, which could be reflected as a larger amplitude in the next recording session. In contrast, a firmly established L1 phonological system may hamper the learning of subtle speech‐sound contrasts from passive L2 exposure. A fact contesting this explanation is that the same nonspeech stimuli have been earlier used in a repeated‐measurement study design in adults, who did not show any amplitude increase (Ylinen et al. 2010). The children's and adults' pattern of results would not, however, be contradictory, if children were more sensitive to this kind of unintended perceptual learning than adults.

Unlike with amplitude, the repeated‐measurement session account does not, however, explain the pattern of results in MMN latency because a significant interaction between the groups and measurement sessions was observed for the latency data. The treatment group showed a decrease of MMN latency, whereas the delayed‐treatment group showed its increase (see Figure 2). The increase of the latency in the delayed‐treatment group is likely due to amplitude increase: Peaking of the larger MMN takes slightly longer. In the treatment group, the MMN had both a larger amplitude and shorter latency, suggesting that here the latency is not modulated by amplitude. Considering the opposite pattern of results in the two groups between the first two measurements, we argue that the decrease of the latency must be due to the treatment, that is, the gaming intervention. This interpretation was further confirmed when the delayed‐treatment group, which was originally assigned to a waiting list, received the gaming intervention. When the effects of the treatment were measured in the 3rd measurement session after they had received the intervention, the MMN latency for the nonspeech sounds had significantly decreased in this group as well, replicating the result of the original treatment group.

The finding that a gaming intervention with a speech‐based language learning game affects the processing of nonspeech sounds that were not trained in the intervention may seem somewhat surprising. During the intervention, the speech‐based language training included speech perception and production as the players had to imitate native English speakers' utterances. This means that to produce the utterances accurately, the children had to focus their attention on the analysis of the quality of L2 speech sounds and words and do auditory‐motor mapping to transform the speech perception into speech production (see Polley et al. 2006, for an animal model of attention effects on auditory perceptual learning). Such active, attentive analysis of the spectral features of speech may attune the processing of sound frequency (Polley et al. 2006), which may also affect pitch processing in nonspeech sounds. Practicing the analysis of the spectral features of speech could then result in a more efficient analysis of sound frequency beyond the speech domain.

Some previous findings are consistent with the current results by showing the effect of training and exposure on MMN latency. Kurkela et al. (2019) exposed adult participants passively to Chinese lexical tones for 8 h. Their results suggest that passive exposure modulated the MMN responses to speech sound changes by shortening the MMN latency. A similar latency effect was observed also for the MMN for nonspeech sounds mimicking the lexical tones used in exposure. Thus, the current results are in line with those of Kurkela et al. (2019). There were, however, some differences between these studies: First, our training was active, whereas that of Kurkela et al. (2019) was passive listening; second, although Kurkela et al. (2019) used nonspeech sounds that mimicked the trained sounds by their pitch contour, the nonspeech sounds used in the current study were not related to the speech sounds trained in the game. In other words, in the current study, the trained speech sounds and the tested nonspeech sounds comprised spectral features, but the frequencies tested were not trained. This means that in the current study, the latency effect observed was generalized to pitch processing beyond the trained sounds and their frequencies.

A possible account for the generalization effect from speech production training to pitch processing is a link between phonology and pitch processing. Pitch is cued by the fundamental frequency of sounds, the processing of which has been shown to be enhanced in early bilinguals: Early exposure to two phonological systems has been suggested to prime the brain to respond to the fundamental frequency (Skoe et al. 2017). Although the current participants certainly were not fully bilingual after short exposure, they nevertheless took steps toward that direction by practicing new L2 sounds, which could, with a longer L2 use, eventually result in a bilingual phonological system. The extension of their phonological system, along with the practice of L2 speech sounds, could, thus, cause some improvement of the pitch processing through the same neural mechanism as in Skoe and colleagues' (2017) study. Along the same lines, previous studies have suggested that pitch processing is associated with phonological awareness in children (Bolduc and Montésinos‐Gelet 2005; Loui et al. 2011; Steinbrink et al. 2019) as well as in adults with congenital amusia (Sun et al. 2017). Pitch processing has also been linked to children's reading performance (Forgeard et al. 2008; Lamb and Gregory 1993), which is dependent on phonological skills. Applying these findings to the current results, our L2 speech training may have improved children's phonological skills and phonological awareness, which could, in turn, influence pitch processing. Together, these findings suggesting a link between different phonological skills and pitch processing are in line with the view that phonological auditory‐motor mapping, which was required to produce the L2 speech sounds and words aloud to proceed in our game, may have improved the children's pitch processing ability in the current study.

Another possible explanation for the pitch processing improvement is that articulation training of new L2 sounds may increase general auditory plasticity, which could in turn be reflected in nonspeech pitch processing. This is based on Simmonds' (2015) hypothesis that articulatory experimentation with new L2 sounds may shift the brain into a learning state that resembles the song‐learning stages observed in songbirds. The current study does not enable us to tease apart these explanations, but there is more experimental evidence on the link between pitch processing and phonological skills in children (Bolduc and Montésinos‐Gelet 2005; Loui et al. 2011; Steinbrink et al. 2019) than on Simmonds' (2015) hypothesis on articulatory learning. Nevertheless, the accounts do not need to be mutually exclusive.

Finally, previous studies have suggested that gaming per se may affect cognitive processes and even auditory processing. A meta‐analysis by Yang et al. (2021) looking at cognitive effects of video game‐based interventions on cognitive function in elderly people has suggested that gaming interventions affect general cognitive scores and processing speed (however, for no significant benefit in the auditory domain, see Powers et al. 2013 and Stewart et al. 2020). The benefits of action video games have also been found for auditory attention and phonological skills (Mancarella et al. 2022). Moreover, Shin et al. (2017) have compared MMN responses to duration changes in nonspeech sounds in frequent players and infrequent players of a mobile puzzle game. The MMN latency was shorter in frequent game players than in infrequent game players, whereas the MMN amplitude was not significantly different between the two groups. Our current results are thus compatible with those of Shin et al. (2017). Although Shin et al. (2017) mention that in their study, the increased processing speed may not be a result of game practice but rather a pre‐existing characteristic of participants who tend to become frequent players, the current study suggests a causal relationship between gaming intervention and processing speed, as indicated by the MMN latency changes in the two groups as a function of training.

Although the decrease of MMN latency was interpreted to be caused by the training, the total length of training in minutes did not correlate with the latency decrease. A possible explanation for the lack of correlation is that the relevant changes may have occurred earlier in the course of the treatment rather than depending on the total length of the training. However, this possibility should be examined more thoroughly in future research.

Despite demonstrating significant effects, the present study also has several limitations. First, the dataset could have been larger both in terms of the number of participants and the amount of data collected per participant. Our sample size may have reduced the sensitivity to detect subtle effects; however, a sensitivity analysis indicated that the sample was sufficient for detecting effects of medium magnitude. Second, the children participating in the current study varied in their English proficiency and level of prior exposure to English; this aspect should be more strictly controlled in future research. Third, the current study did not include behavioral validation measures that could have directly linked neural changes to improvements in behavioral discrimination performance. Behavioral measures are not always reliable indicators of discrimination skills in children, given their limited attention spans, difficulties in following task instructions, and high variability in motivation (see Ylinen et al. 2021). Nonetheless, future studies with larger samples and behavioral outcome measures will help to strengthen and extend the present findings. Furthermore, because we observed test–retest effects in children's MMN amplitude, such studies should also explicitly control for test–retest influences.

In sum, previous findings suggest that the current effect of improved pitch processing may be due to improved phonological skills, including the extension of the phonological system by L2 sounds as a result of training (see Skoe et al. 2017) or the improvement of phonological awareness (Bolduc and Montésinos‐Gelet 2005; Loui et al. 2011; Steinbrink et al. 2019). In addition, L2 training may also increase general auditory plasticity (Simmonds 2015), or the MMN latency shortening may be due to gaming that was used as the method of intervention (Shin et al. 2017). It is also possible that all of these factors drive the accelerating effect on pitch processing. If both auditory‐motor task type and gaming with feedback seem to drive similar plastic changes in the brain, a game‐based approach may be particularly well‐suited for rehearsing phonological (auditory‐motor) skills. In addition to the game per se, feedback given by its speech recognizer likely enhanced the learning effects by motivating the children to try their best during speech rehearsal and by drawing the children's attention to the sounds rehearsed (for comparison of learning effects in the game with feedback vs. nongame with no feedback, see Junttila et al. 2022).

## 5 Conclusion

The current study demonstrates that, as indicated by the MMN latency, game‐based L2 speech rehearsal involving auditory–motor mapping between speech perception and production may improve pitch processing in the brain, even for nonspeech sounds that were not part of the training. This suggests that second‐language speech training can enhance neural processing of untrained auditory stimuli, thereby demonstrating cross‐domain auditory plasticity.

## Author Contributions

**Sari Ylinen:** conceptualization, formal analysis, funding acquisition, methodology, project administration, resources, supervision, validation, visualization, writing – original draft, writing – review and editing. **Katja Junttila:** data curation, formal analysis, investigation, writing – review and editing. **Anna‐Riikka Smolander:** investigation, methodology, resources, writing – review and editing. **Reima Karhila:** methodology, software, writing – review and editing. **Mikko Kurimo:** funding acquisition, methodology, project administration, resources, software, writing – review and editing.

## Funding

This work was supported by the Research Council of Finland (274058 and 274075) and NordForsk (103893).

## Conflicts of Interest

The authors declare no conflicts of interest.

## Supporting information




**Data S1:** Supporting information.

## Data Availability

The data reported in this study are not publicly available due to the lack of permission from participants and their guardians for open dissemination. However, anonymized data may be made available by the authors upon reasonable request and with appropriate ethical approval.
