# Supplementary material for: Game‐Based Foreign‐Language Speech Rehearsal Improves Pitch Processing Beyond Speech Domain
Source: Eur J Neurosci. 2026 Jul 6;64(1):e70603. doi: 10.1111/ejn.70603 (PMC13334341; doi:10.1111/ejn.70603)

Supplementary materials

Supplementary material 1: ERP responses to pitch standards and deviants in the treatment group

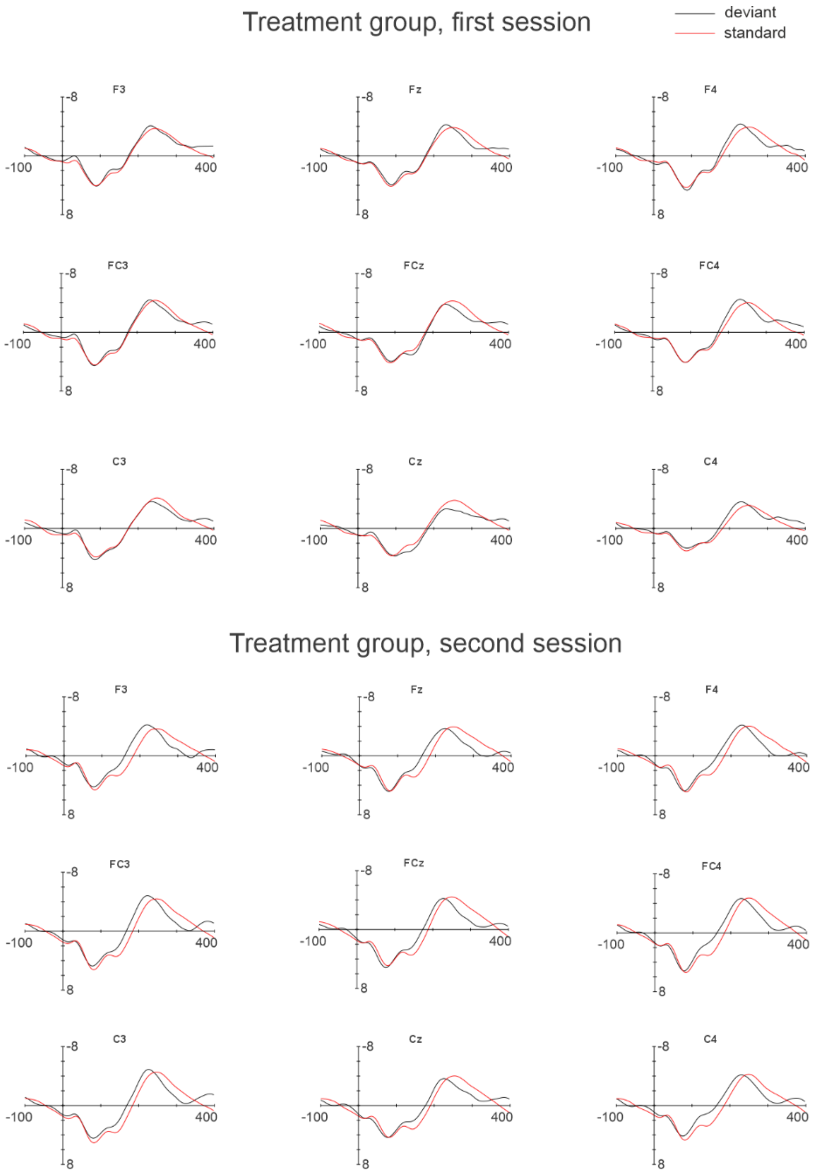

Supplementary material 2: ERP responses to pitch standards and deviants in the delayed-treatment group

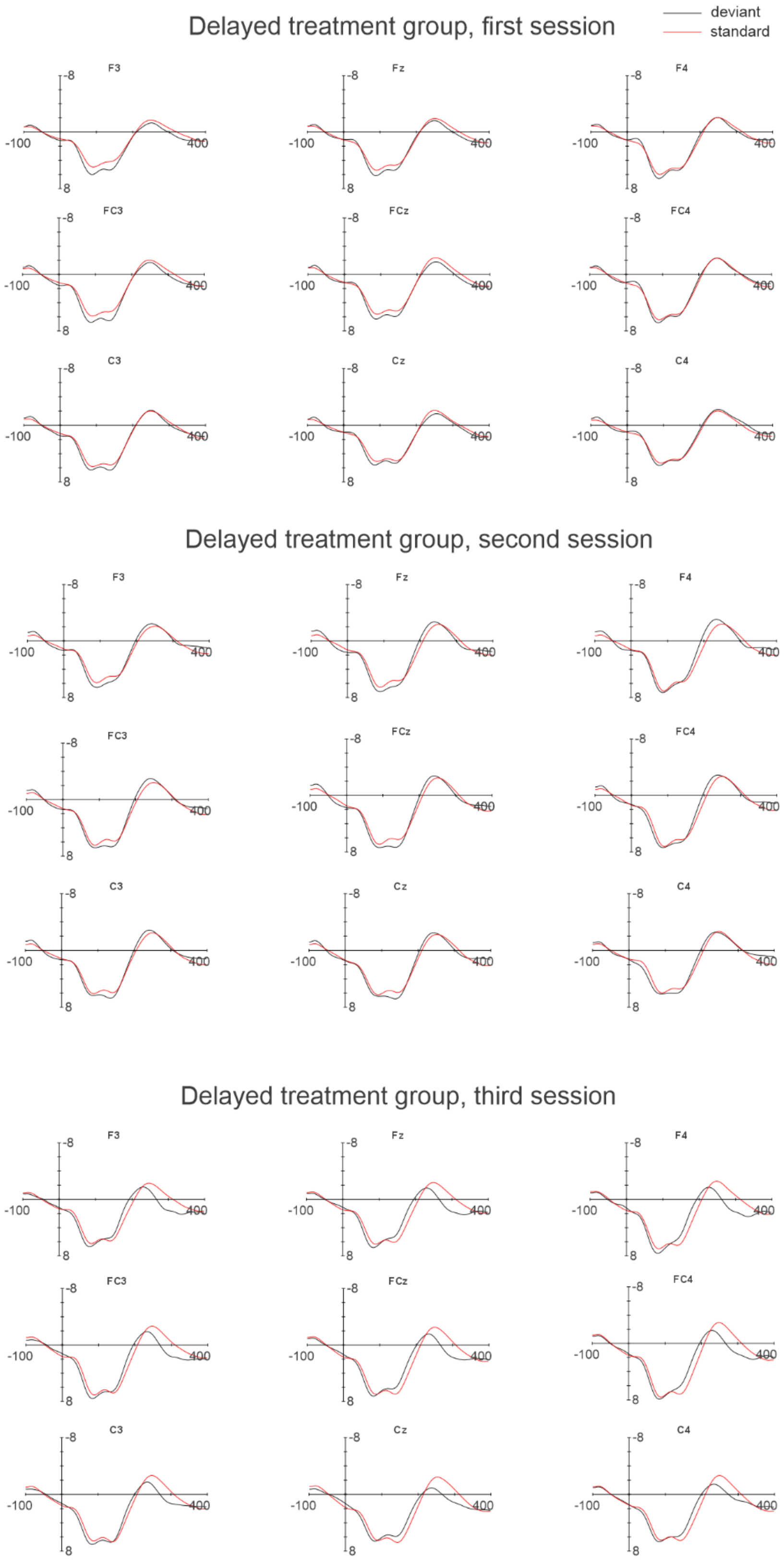

Supplementary material 3: MMN responses (deviant-minus-standard difference waveforms) to duration deviants in the treatment and delayed-treatment groups

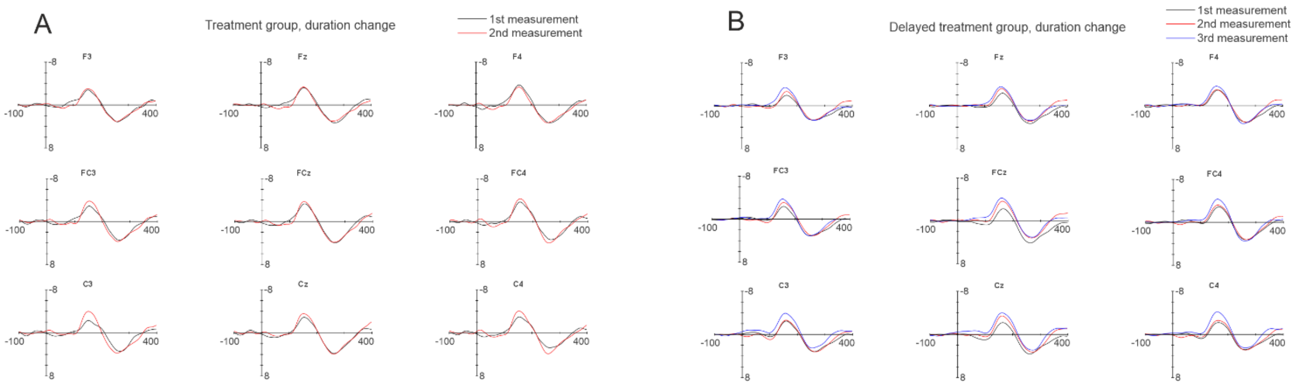

Supplement: Supplementary file 1 — Data S1: Supporting information. [file EJN-64-0-s001.pdf]
